# Experimental and Computational Observations of Immunogenic Cobalt Porphyrin Lipid Bilayers: Nanodomain-Enhanced Antigen Association

**DOI:** 10.3390/pharmaceutics13010098

**Published:** 2021-01-14

**Authors:** Jasmin Federizon, Conrard Giresse Tetsassi Feugmo, Wei-Chiao Huang, Xuedan He, Kazutoyo Miura, Aida Razi, Joaquin Ortega, Mikko Karttunen, Jonathan F. Lovell

**Affiliations:** 1 Department of Biomedical Engineering, University at Buffalo, State University of New York, Buffalo, NY 14260, USA; jasminfe@buffalo.edu (J.F.); weichiao@buffalo.edu (W.-C.H.); xuedanhe@buffalo.edu (X.H.); 2 Department of Chemistry, the University of Western Ontario, London, ON N6A 3K7, Canada; conrardgiresse.tetsassifeugmo@nrc-cnrc.gc.ca; 3 Laboratory of Malaria and Vector Research, National Institute of Allergy and Infectious Diseases, National Institutes of Health, Rockville, MD 20852, USA; kmiura@niaid.nih.gov; 4 Department of Anatomy and Cell Biology, McGill University, Montreal, QC H3A 0C7, Canada; aida.razi@mail.mcgill.ca (A.R.); joaquin.ortega@mcgill.ca (J.O.); 5 Centre for Advanced Materials and Biomaterials Research, the University of Western Ontario, London, ON N6A 3K7, Canada; 6 Department of Applied Mathematics, the University of Western Ontario, London, ON N6A 5B7, Canada

**Keywords:** molecular dynamics, simulations, bilayers, antigens, particles, vaccines, malaria

## Abstract

Cobalt porphyrin phospholipid (CoPoP) can incorporate within bilayers to enable non-covalent surface-display of antigens on liposomes by mixing with proteins bearing a polyhistidine tag (his-tag); however, the mechanisms for how this occurs are poorly understood. These were investigated using the his-tagged model antigen Pfs25, a protein antigen candidate for malaria transmission-blocking vaccines. Pfs25 was found to associate with the small molecule aquocobalamin, a form of vitamin B12 and a cobalt-containing corrin macrocycle, but without particle formation, enabling comparative assessment. Relative to CoPoP liposomes, binding and serum stability studies indicated a weaker association of Pfs25 to aquocobalamin or cobalt nitrilotriacetic acid (Co-NTA) liposomes, which have cobalt displayed in the aqueous phase on lipid headgroups. Antigen internalization by macrophages was enhanced with Pfs25 bound to CoPoP liposomes. Immunization in mice with Pfs25 bound to CoPoP liposomes elicited antibodies that recognized ookinetes and showed transmission-reducing activity. To explore the physical mechanisms involved, we employed molecular dynamics (MD) simulations of bilayers containing phospholipid, cholesterol, as well as either CoPoP or NTA-functionalized lipids. The results show that the CoPoP-containing bilayer creates nanodomains that allow access for a limited but sufficient amount of water molecules that could be replaced by his-tags due to their favorable free energy properties allowing for stabilization. The position of the metal center within the NTA liposomes was much more exposed to the aqueous environment, which could explain its limited capacity for stabilizing Pfs25. This study illustrates the impact of CoPoP-induced antigen particleization in enhancing vaccine efficacy, and provides molecular insights into the CoPoP bilayer properties that enable this.

## 1. Introduction

Subunit vaccines formed from recombinant antigens hold potential for controlling infectious diseases such as malaria [1]. Biomaterials that have structural versatility and biocompatibility can be designed as adjuvants and antigen carriers to augment immune responses [2,3,4,5,6]. Liposomes, which are self-assembling phospholipid bilayer vesicles, hold potential for vaccine adjuvants that also incorporate immunostimulant molecules, as has been demonstrated with the clinical efficacy of the Adjuvant System 01 (AS01) and the Army Liposome Formulation (ALF) formulations, for example [7]. They can improve antigen delivery and presentation to antigen-presenting cells and improve immune cell infiltration and maturation [8,9]. The adjuvant effect can benefit from physical or chemical association of antigens through conjugation to vesicle surface or encapsulation within the aqueous core [10]. The surface functionalization approach provides antigen accessibility on the liposomal surface for antibody or B cell receptor recognition [11].

An incorporation strategy involving non-covalent attachment of antigens on the surface of metallo-chelating liposomes using a polyhistidine tag (his-tag) as anchor allows natural presentation of antigenic epitopes on the liposomal surface without chemical modification [12]. Porphyrins are well-suited for metallochelation strategies [13]. One unique strategy is exemplified by a liposomal vaccine platform containing cobalt porphyrin phospholipid (CoPoP), of which the spatial location inside the hydrophobic bilayer is thought to be responsible for improved his-tag protein binding stability in physiological conditions, compared to nickel-chelating liposomes [14]. Spontaneous antigen attachment to pre-formed CoPoP liposomes is thought to occur by insertion of a his-tag into the bilayer membrane and subsequent coordination of the imidazole moiety to the metal center, while the antigen itself is displayed on the liposome surface. While intuitively plausible, such a mechanism has not been accounted for. From the physical point of view, one of the main questions is how the his-tag accesses the bilayer interior since liposomes (especially those used for drug delivery) can be tightly packed gel state with minimal access to the membrane interior [15]. Here, we attempt to answer this question by coupling atomistic MD simulations with experiments.

A recent study demonstrated the potent vaccine adjuvant effects of CoPoP/PHAD (Phosphorylated HexaAcyl Disaccharide; synthetic monophosphoryl lipid A) liposomes in inducing durable functional antibodies against *Plasmodium falciparum* surface protein (Pfs25), a malaria transmission-blocking vaccine antigen candidate [16]. Previously, antigen-engineering has been pursued to improve the induction of antibodies against Pfs25, including the use of gold [17] or polymer [18] nanoparticles, and the use of virus-like particles (VLPs) [19]. Other approaches make use of recombinant protein engineering for downstream Pfs25 multimerization [20] or attachment to VLPs [21]. The CoPoP system has been demonstrated for other his-tagged antigens related to malaria transmission-blocking [22,23] and other infectious diseases [24,25,26].

**Figure 1:**
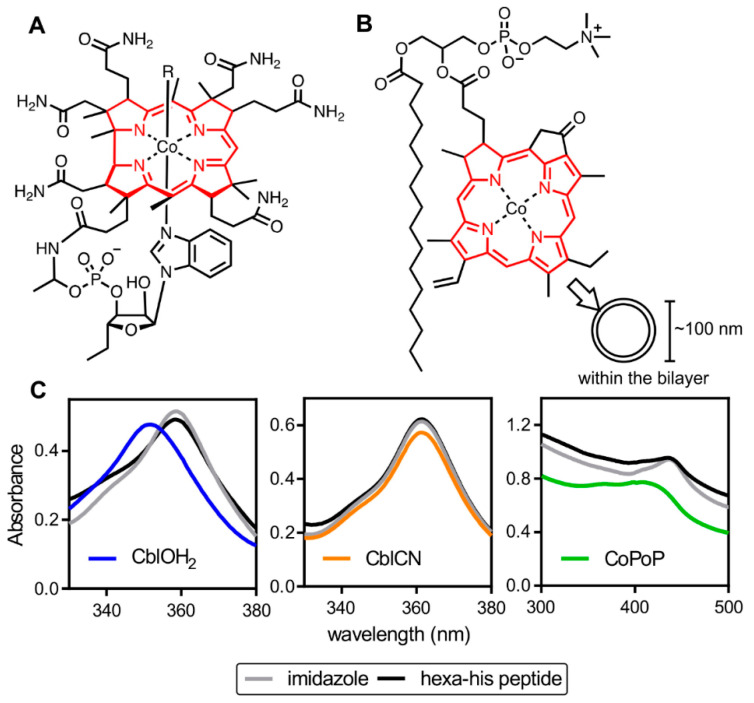
Cobalt tetrapyrroles and spectral shifts induced by imidazoles. Structure of aquocobalamin (**A**) and cobalt porphyrin phospholipid (CoPoP) (**B**). Porphyrin and corrin rings are rendered in red. (**C**) Absorption spectra of aquocobalamin, cyanocobalamin, and CoPoP liposomes before and after incubation with imidazole or a his-tagged peptide. Spectral data shown are the averages of three independent scans.

Vesicle size is a physical property that can play a role in the adjuvanticity of a particulate delivery system [27,28,29,30]. It affects clearance at the injection site, trafficking to lymph nodes, and consequently downstream immune responses. This study investigates the importance of particleization in cobalt tetrapyrrole immunization. Since CoPoP will naturally form nanostructures in aqueous buffers, aquocobalamin (CblOH_2_), which is a water-soluble tetrapyrrole macrocycle containing cobalt, was utilized to mimic the antigen attachment method in a soluble and non-particleized form. The core macrocycle in aquocobalamin is corrin, a porphyrin-related ring consisting of four reduced pyrrole rings linked by three –CH = methylene bridges and one direct bond (Figure 1). We complement experiments by performing MD simulations to study the differences in the microscopic interactions in the CoPoP, solvated cobalamin and Co-NTA liposome systems.

## 2. Materials and Methods

### 2.1. Liposome Preparation and Characterization

CoPoP liposomes composed of a mass ratio of 4:2:1 DPPC (Corden # LP-R4-057):cholesterol (PhytoChol, Wilshire Technologies, Princeton, NJ, USA):CoPoP (synthesized as previously described [14]), prepared by ethanol injection and nitrogen-pressurized lipid extrusion. Extruded liposomes were dialyzed in phosphate buffered saline (PBS) at 4 °C to facilitate removal of ethanol and then were characterized by dynamic light scattering (DLS) using a NanoBrook 90Plus PALS instrument to measure liposome size and polydispersity index. CoPoP concentration was adjusted to 320 µg/mL by dilution.

### 2.2. Absorption Spectra of Cobalt Tetrapyrroles

Imidazole (Sigma-Aldrich, St. Louis, MO, USA, # I202) and his-tagged peptide (with the amino acid sequence SELLSLINDMPITNDQKKLMSHHHHHH with no internal histidine residue, synthesized by Genscript) were incubated with hydroxocobalamin hydrochloride (Sigma-Aldrich # 95200), cyanocobalamin (Sigma-Aldrich # V2876), and CoPoP liposomes at a final concentration of 0.5 mM overnight at 4 °C. Pre- and post-incubation absorption spectra of aquocobalamin, cyanocobalamin, and CoPoP liposome were acquired using Perkin Elmer UV-Vis spectrophotometer Lambda 365 at 0.5 nm intervals.

### 2.3. Characterization of Histidine Binding to Cobalt Tetrapyrroles

His-tagged Pfs25, prepared from a Baculovirus system as previously described [31], was diluted to 80 µg/mL and incubated with varying amounts of hydroxocobalamin hydrochloride. At the optimum binding ratio, hydroxocobalamin hydrochloride (320 µg/mL) was incubated with 80 µg/mL Pfs25 at different time points and then subjected to native electrophoresis using Tris-Glycine gel (Lonza, Basel, Switzerland). Hydroxocobalamin acetate (Sigma-Aldrich # H8017) and cyanocobalamin were also incubated with Pfs25 for comparison at 1:4 Pfs25:Cbl/CoPoP binding ratio. For peptide binding, a fluorescently labeled peptide, FAM-RGD, was used and was previously demonstrated to bind to CoPoP liposomes [14].

### 2.4. Cryo-Electron Microscopy (Cryo-EM)

CoPoP liposomes (320 µg/mL) were mixed with his-tagged Pfs25 (80 µg/mL) at an equal volume ratio for 3 h at room temperature. The formulation also included PHAD (320 µg/mL). Holey carbon grids (c-flat CF-2/2-2C-T) were washed with chloroform overnight and glow-discharged at 5 mA for 15 s immediately before sample application. Approximately 3.6 µL of the reaction mixture was deposited on EM grid. Vitrification was performed using a Vitrobot (ThermoFisher, Waltham, MA, USA) by blotting the grids once for 3 **s** and blot force +1 before they were plunged into liquid ethane. Temperature and relative humidity in the Vitrobot chamber during the vitrification process were maintained at 25 °C and 100%, respectively. Grids were loaded using a Gatan 626 single tilt cryo-holder and introduced into a Tecnai F20 electron microscope (ThermoFisher) operated at 200 kV and equipped with a Gatan K2 Summit direct detector device camera (Gatan, Pleasanton, CA, USA). This detector was used in counting movie mode with five electrons per pixel per second for 15 s exposures and 0.5 s/frame. This method produced movies consisting of 30 frames with an exposure rate of ~1 e^−^**/**Å^2^. Movies were collected with a nominal defocus of −2.5 µm and a nominal magnification of 25,000×, which produced images with a calibrated pixel size of 1.45 Å. Motion correction for these images was done using MotionCor2 [32]. Images were trimmed and prepared for figures using Photoshop (Adobe, San Jose, CA, USA)

### 2.5. Fluorescence Quenching of Proteins and Peptides

Prior to labeling with DY-490-NHS-Ester (Dyomics, Jena, Germany, # 490-01), Pfs25 was dialyzed at 4 °C against sodium bicarbonate solution pH 9.0 at least twice. Stock solution of the dye was added at five-fold molar excess to the dialyzed sample, followed by stirring at room temperature for 2 h. Extensive dialysis against PBS was then performed to remove any free dye. Post-dialysis protein concentration was quantified using a micro-BCA assay. Liposomes (160 µg/mL CoPoP or CoNTA), or aquocobalamin (1600 µg/mL) were incubated with FAM-RGD peptide or labeled protein (40 µg/mL), and fluorescence measurements were acquired at excitation and emission wavelengths of 495 and 525 nm, respectively, on a 5 nm bandwidth using TECAN Safire multi-plate reader. Percentage fluorescence quenching was calculated by comparing to the free peptide or protein. Fetal bovine serum (ThermoFisher # 10438026) was added to a final concentration as indicated.

### 2.6. Enzyme-Linked Immunosorbent Assay (ELISA)

Anti-Pfs25 IgG titers were estimated by ELISA. A 96-well plate (Thermo Scientific Nunc, # 442404) was coated with 100 ng/well Pfs25, blocked with 2% bovine serum albumin (BSA) in PBS containing 0.1% (*w*/*v*) Tween-20 (PBS-T), and incubated with mouse serum serially diluted in 1% BSA in PBS-T. After incubation with horseradish peroxidase-conjugated goat anti-mouse IgG secondary antibody (Genscript, Piscataway, NJ, USA, # A00160), tetramethylbenzidine (Amresco, Dallas, TX, USA, # J644) was added. After color development, 1M HCl was added to stop the reaction. Absorbance at 450 nm was acquired using TECAN Safire multi-plate reader, (TECAN, Mannedorf, Switzerland). Endpoint titers were defined as the reciprocal serum dilution that produced an absorbance cutoff of 0.5.

### 2.7. Antigen Uptake Study

RAW264.7 murine macrophage cells (ATCC, Manassas, VA, USA, # TIB-71^TM^) were cultured in a 24-well plate in Dulbecco’s Modified Eagle’s Medium (DMEM, ThermoFisher Scientific) containing 10% fetal bovine serum, 1% penicillin/streptomycin and grown to a confluence of approximately 70–80%. Macrophage cells were incubated for 4 h at 37 °C with the indicated samples at a final concentration of 1 µg/mL DY-490-conjugated Pfs25. Following incubation, macrophage cells were re-suspended in PBS and subjected to flow cytometry using BD LSRFortessa X-20 flow cytometer. FlowJo (version 10, FlowJo, Ashland, OR, USA) software was used for data analysis.

### 2.8. Murine Immunization

Animal experiments were conducted in accordance with the University at Buffalo Institutional Animal Care and Use Committee (IACUC) regulations, protocol # BME05044. Female eight-week-old CD-1^®^ (ICR) mice received intramuscular (IM) injections containing 250 ng of Pfs25 combined with indicated adjuvants on days 0 and 21. Mouse sera were collected on day 42. CoPoP liposomes were incubated with Pfs25 at 4:1 mass ratio of CoPoP:protein for 3 h at room temperature prior to injection and diluted in PBS to achieve the desired antigen dose for immunization. For Alhydrogel^®^ adjuvant, 2% aluminum gel (Accurate Chemical and Scientific Corporation, Westbury, NY, USA, # A1090BS), alum was mixed with the antigen to a final concentration of 1.5 mg/mL.

### 2.9. Antibody Analysis

For Indirect Immunofluorescence Assay (IFA)*, P. falciparum* ookinetes were obtained from the midgut of *Anopheles stephensi* mosquitoes and fixed onto slides as previously described [16]. After blocking with 5% BSA in PBS-T, slides were incubated with mouse serum diluted 1/500 in 5% BSA in PBS at 37 °C for 1 hr and subsequently washed with PBS in a humidity chamber for 5 min thrice. DyLight488-conjugated goat anti-mouse IgG secondary antibody (ImmunoReagents, Raleigh, NC, USA, # GtxMu-003-F488NHSX) was diluted 1/500 in 5% BSA in PBS and then incubated with slide for 30 min at 37 °C. Slides were mounted with ProLong Gold Antifade with 4′,6-diamidino-2-phenylindole (DAPI, Thermo Fisher Scientific # P36931) and imaged with an EVOS FL microscope using a 100× objective lens. For Standard Membrane-Feeding Assay (SMFA), IgG was purified from pooled mouse serum (*n* = 4) via protein G affinity chromatography (Pierce, Rockford, IL) and mixed with a mature gametocyte culture of *P. falciparum* NF54 to a final concentration of 0.75 mg/mL. Using a membrane-feeding apparatus, the final mixture was fed to female *Anopheles stephensi* (Nijmegen strain) mosquitoes. After 8 days, the mosquitoes (*n* = 20 per sample) were dissected to enumerate the oocysts in the midguts.

### 2.10. Molecular Dynamics Simulations

All-atom molecular dynamics (MD) simulations were carried out in an aqueous solution. Four different systems were simulated: (1) water + cyanocobalamin, (2) water + dipalmitoylphosphatidylcholine (DPPC) + cholesterol (termed pure bilayer here), (3) water + DPPC + Cholesterol + CoPoP (functionalized bilayer), and (4) water + DPPC + Cholesterol + NTA (functionalized bilayer). The cobalamin model used here has its basis on Marques et al. [33] who used experimental data from 22 cobalamin structures including both cyano- and aquoacobalamin. They noted that only the inclusion of complex alkyl ligands (R in Figure 1A) required additional structural optimization of the bond properties. As Marques et al. describes, the use of cyanocobalmin can be justified when structural properties are the main interest. The bilayer models were generated using the CHARMM-GUI Membrane Builder [34], and the CHARMM36 force field was used [35]. The CHARMM36-compatible TIP3P water model was used for water [36,37]. All the systems were built in a rectangular box containing a total of 512 lipids (256 per leaflet) with a hydration number of 40 water molecules per lipid. The ternary systems were composed of 45% DPPC, 45% cholesterol (CHL), and 10% of CoPoP or NTA. Partial atomic charges were computed with an electrostatic potential (ESP) fitting approach. The MD simulations were carried out using GROMACS 2018 package [38].

The systems were first energy minimized using the steepest descents algorithm followed by six steps of equilibration runs. The first two steps composed simulations in the constant number of atoms, volume, and temperature (NVT) ensemble and the remaining ones in the NPT (constant atoms, pressure, and temperature) ensemble. At least 400 ns was used for the production runs in the NPT ensemble at 298.15 k and 1 bar. The V-rescale thermostat was used for temperature coupling [39]. For pressure coupling, the Parrinello–Rahman [40] method was used with semi-isotropic coupling. The simulations were carried out using periodic boundary conditions (PBC) and the particle-mesh Ewald (PME) method for electrostatic interactions [41]. A force-switch cutoff approach was used for the Lennard-Jones potential over 1.0–1.2 nm. The Parallel Linear Constraint Algorithm (P-LINCS) [42,43] algorithm was used to restrain the bond lengths. A time step of 2 fs was used, and data were saved every 10.0 ps.

## 3. Results and Discussion

### 3.1. Experimental Results

Analogous to the porphyrin ring, the structure of cobalamins (vitamin B12) features a central cobalt atom that is tightly coordinated to the four equatorial nitrogen donors from the corrin ring (Figure 1A). The heterocyclic nitrogen atom of 5,6-dimethylbenzimidazole occupies the lower α-axial fifth coordination site in the base-on configuration whereas the upper β-axial position (sixth) may be occupied by various ligands, i.e., H_2_O in aquocobalamin [44]. The axial β-ligand in aquocobalamin is exchangeable and hence can be displaced by the imidazole group, which exhibits intermediate affinity [45,46]. This β-ligand exchange in the octahedral complex allows attachment of Pfs25 to aquocobalamin via coordinate bonding of the imidazole nitrogen of any histidine residue to the metal center. Displacement of the aquo ligand by the histidine residue, however, is not exclusive to the purification tag. Due to favorable accessibility in the upper plane of aquocobalamin, binding of any exposed histidine residue on the protein surface to aquocobalamin is possible. In contrast, CoPoP binding necessitates a short segment of histidine residues to facilitate insertion into the hydrophobic bilayer prior to coordination [14]. Importantly, the mode of binding in aquocobalamin exemplifies a close representation of CoPoP binding in aqueous medium. Moreover, the non-toxic nature of aquocobalamin eliminates the possibility of any undesired side effects upon vaccination. IM injection of high doses of cyanocobalamin did not elicit adverse reactions in humans [47].

Imidazole coordination to the cobalt center is reflected by the changes in the absorption spectrum. Post-incubation absorption spectra for aquocobalamin, but not cyanocobalamin (CblCN), revealed a spectral shift towards longer wavelength (Figure 1C). The red shift has also been observed in a binding study with transcobalamin, an aquocobalamin-binding protein [46]. The absence of a bathochromic shift in cyanocobalamin indicates that the cyanide group was not substituted by imidazole, which is likely due to the strong metal–carbon bond. Coordination of the imidazole to the metal center in CoPoP liposome results in a bathochromic shift in absorption.

**Figure 2:**
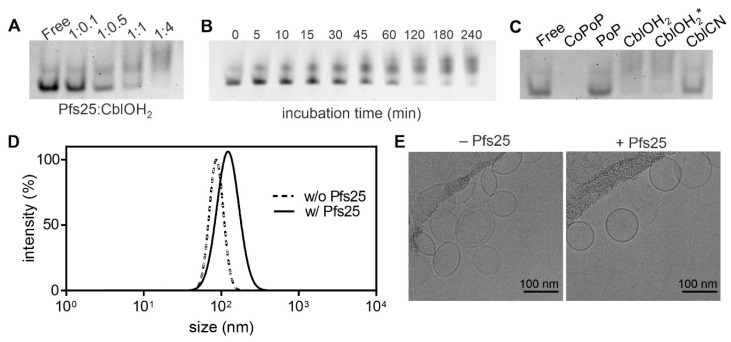
Binding characteristics of his-tagged Pfs25 with cobalt tetrapyrroles. (**A**) Binding mass ratio of Pfs25 to aquocobalamin as evaluated by native PAGE. The indicated ratio of antigen to aquocobalamin is indicated, or is labeled as “Free” for the protein alone. (**B**) Kinetics of Pfs25 binding to aquocobalamin incubated at 1:4 mass ratio at room temperature. (**C**) Pfs25 binding to different cobalt tetrapyrroles under the same binding conditions. * hydroxocobalamin acetate. (**D**) Representative plot of CoPoP liposome size against intensity based on dynamic light scattering before and after incubation with Pfs25. (**E**) Cryo-electron micrographs of CoPoP liposomes with and without bound Pfs25. Pfs25 was incubated with CoPoP liposomes for 3 h before acquisitions using dynamic light scattering (DLS) and cryo-EM.

Association of Pfs25 to the aquocobalamin complex is associated with changes in the electrophoretic migratory properties that arise from the small size increase and different overall charge. Applying the same binding conditions to aquocobalamin resulted in an optimum binding mass ratio similar to CoPoP (Figure 2A), which is 1:4 antigen:CoPoP/Cbl. The 1:4 ratio of antigen: CoPoP when incubating Pfs25 with CoPoP liposomes was previously found to be required to ensure full protein binding [16]. Deviation from the expected stoichiometry of the substitution reaction for aquocobalamin can be partly attributed to the fact that hydroxocobalamin hydrochloride is a mixture of CblOH and CblOH_2_ under physiological conditions [48].

Binding kinetics (Figure 2B) showed that binding of his-tagged protein to aquocobalamin started to plateau about 3 h after incubation. Ligand substitution was also observed for hydroxocobalamin acetate, an analog of hydroxocobalamin hydrochloride. Cyanocobalamin with the tightly bound β-group displayed no apparent band shift in native electrophoresis (Figure 2C), suggesting non-association of Pfs25. CoPoP and PoP liposomes serve as positive and negative controls, respectively. Dynamic light scattering measurements depicted a slight increase in liposomal size and polydispersity after binding of Pfs25 (Figure 2D). The charge of these liposomes was previously shown to be negative, before or after Pfs25 binding [16]. Cryo-electron micrographs indicate that liposomal shape remains predominantly unaltered after binding (Figure 2E). Some CoPoP liposomes, prior to protein binding, appeared to have a slight almond shape, for reasons that are unclear. The 25 kDa size of the Pfs25 is too small to be directly visualized by cryo electron micrographs.

**Figure 3:**
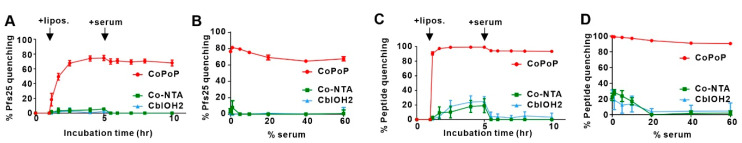
Relative stability of cobalt tetrapyrrole complexes in buffer with and without serum. Binding was assessed with a fluorescence quenching assay. The percentage of fluorescence quenching was calculated based on the intensity of the free peptide or protein. Error bars represent standard deviations for *n* = 3 experiments. (**A**) Quenching of labeled Pfs25 as it is first incubated with liposomes or aquocobalamin and then after 20% bovine serum is added. (**B**) Quenching of labeled Pfs25 after it is bound to various forms of cobalt and then incubated with bovine serum as indicated. (**C**) Quenching of a fluorescent peptide as it is first incubated with liposomes or aquocobalamin and then after 20% bovine serum is added. (**D**) Quenching of a fluorescent peptide after it is bound to various forms of cobalt then incubated with bovine serum as indicated.

When fluorescently-labeled Pfs25 was incubated with CoPoP liposomes, strong quenching of the fluorescence was observed (Figure 3A). This is due to fluorescence energy transfer from the dye to the CoPoP in the bilayer upon binding. In contrast, fluorescent quenching of Pfs25 to CoNTA liposomes or aquocobalamin was limited. After 20% serum addition, most of the quenching remained intact with Pfs25 bound to CoPoP liposomes. The small amount of quenching was disrupted for CoNTA and aquocobalamin. Aquocobalamin, which is a small molecule chromophore, may be limited to quench the fluorescently-labeled protein relative to the dense CoPoP bilayer. The fluorescent quenching of Pfs25 bound to CoPoP liposomes remained intact in varying concentrations of sera (Figure 3B). A similar trend was observed with a fluorescently labeled peptide (Figure 3C), although the amount of fluorescent quenching was greater with CoNTA and aquocobalamin. This may be due to the smaller size of the peptide and closer proximity of the fluorophore to the macrocycle. With serum addition (Figure 3C), and in the presence of serum (Figure 3D), the quenching was disrupted for CoNTA and aquocobalmin, but not for CoPoP. Competition might have arisen from non-specific proteins in serum or cobalamin-binding proteins in serum such as transcobalamin II [49]. Poor serum stability of the cobalamin-antigen and CoNTA complex reflects weak association of the antigen through non-covalent interactions in the aqueous mileu, suggesting the advantage of the sheltered antigen binding site in CoPoP liposome. This is discussed further below in connection with MD simulations.

**Figure 4:**
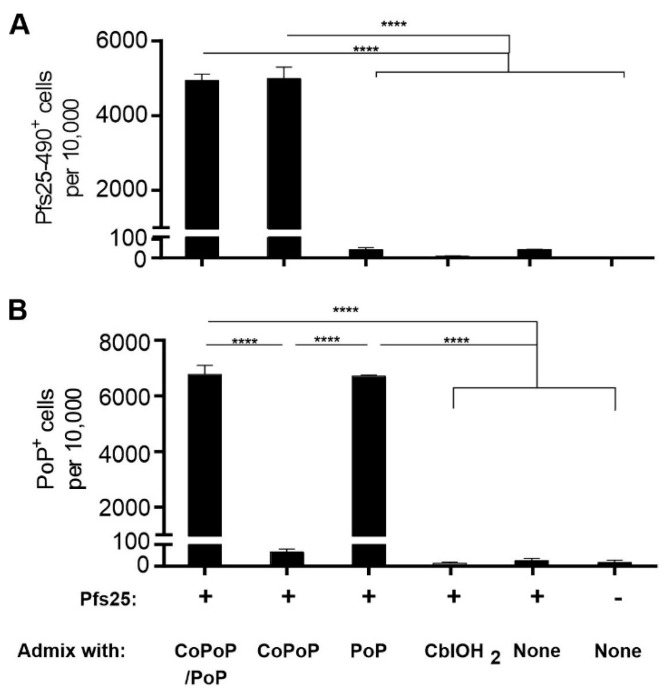
Antigen-cobalt tetrapyrrole uptake in macrophages. Pfs25-Dy490 (**A**) and PoP (**B**) uptake by murine RAW 264.7 macrophage cells following 4 hr incubation with indicated samples and Pfs25. CoPoP and cobalamin have minimal PoP fluorescence. Error bars represent mean +/− standard deviation for *n* = 3 experiments. **** *p* < 0.0001, analyzed by one-way ANOVA with Bonferroni multiple comparisons post-test.

An antigen uptake study was carried out using murine macrophages, as model antigen presenting cells, using the fluorescently labeled antigen and flow cytometry with the gating strategy shown in Figure S1 of the Supporting Information. While antigen uptake was minimal when Pfs25 was in its free form, efficient cellular internalization was observed when Pfs25 was liposome-bound (Figure 4). Given the instability of cobalamin-antigen complex in serum, it was expected that Pfs25 uptake would be similar to that for the free form. Likewise, minimal antigen internalization was observed for the non-chelating PoP liposomes. High liposome internalization was obtained for PoP liposomes, but not for CoPoP liposomes due to low fluorescence signal. To augment fluorescence of CoPoP liposomes, CoPoP/PoP liposomes containing 1:1 ratio of CoPoP:PoP was produced. With CoPoP/PoP liposomes, high antigen and liposome internalization were observed.

**Figure 5:**
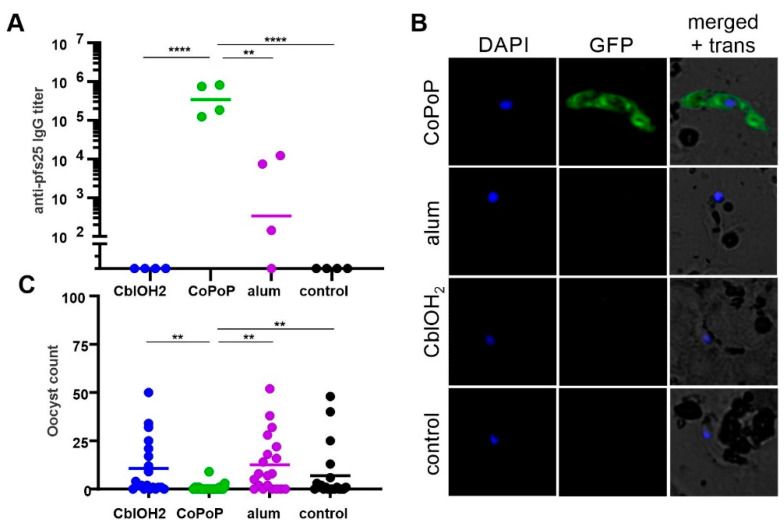
Immunogenicity of his-tagged Pfs25 and functionality of induced antibodies. (**A**) ELISA titers following prime-boost vaccination of CD-1 mice with 250 ng of Pfs25 bound to indicated samples. (**B**) Immunofluorescence assay of fixed *P. falciparum* ookinetes using goat anti-mouse IgG (H + L) secondary antibody DyLight^®^ 488 conjugate. Mice sera and secondary antibodies were both diluted at 1:500. (**C**) Transmission-reducing activity based on oocysts intensity of pooled mouse sera antibodies. Horizontal bars in (**A**,**C**) represent the geometric mean for *n* = 4 mice and the arithmetic mean for *n* = 20 mosquitoes, respectively. Average oocyst counts are indicated above. Asterisks denote statistical significance for the CoPoP group in (**A**) as assessed by one-way ANOVA with Bonferroni multiple comparisons post-test comparisons of the log-transformed data and in (**C**) as assessed by using a zero-inflated negative binomial model with Bonferroni correction; ** *p* < 0.01 and **** *p* < 0.0001.

Pfs25 is a sexual stage protein expressed on the surface of zygote and ookinete forms of *P. falciparum* [50,51]. At 250 ng antigen dose, immunogenicity of three delivery platforms of varying size was evaluated using enzyme-linked immunosorbent assay (ELISA) (Figure 5A). Prime-boost immunization with Pfs25 bound to 100-nm liposomes on days 0 and 21 elicited an antibody titer on the order of 10^5^, which is at least two orders of magnitude higher than that for the micrometer-sized alum. Vaccination with aquocobalamin, which is presumably less than 10 nm in size, failed to induce Pfs25-specific antibodies. Immunofluorescence micrographs revealed that Pfs25-specific antibodies induced by CoPoP liposome immunization can recognize surface epitopes on ookinetes (Figure 5B). No apparent labeling of ookinetes observed for antisera raised against alum suggests low levels of functional antibodies. Poor immunogenicity of aquocobalamin-bound Pfs25 is substantiated by the absence of surface-labeled ookinetes.

Malaria transmission-blocking vaccines based on Pfs25 work by inducing antibodies that inhibit parasite development within the vector, Anopheles mosquitoes. Assessment of transmission blocking activity of mouse sera antibodies induced by CoPoP liposome vaccination by SMFA demonstrated the capacity of Pfs25-specific IgG antibodies to prevent oocyst formation (Figure 5C). SMFA studies further showed no functionality of antibodies from immunization with cobalamin-bound and alum-adsorbed Pfs25. Poor potency of cobalamin-antigen complex, the non-particleized form, to induce functional antibodies is probably due to its small dimension and loose antigen association. This highlights the crucial role of particle stability in cobalt tetrapyrrole immunization.

### 3.2. Simulation Results

Molecular dynamics (MD) simulations were used to examine the atomic-level mechanisms that account for the observations that CoPoP could bind his-tagged antigens to form stable particles, whereas Co-NTA and aquocobalamin could not. Four systems, a reference bilayer with DPPC and Chol only, a CoPoP functionalized bilayer, an NTA functionalized bilayer and cyanocobalamin in aqueous solution, were studied by MD simulations. The CoPoP and NTA systems were used to examine the underlying microscopic mechanisms and the cyanocobalamin system was used as a comparator. The last 10 ns of the 400 and 500 ns production runs were used for analysis. All systems were determined to be in equilibrium by examining the convergence of the area per lipid versus time as well as other structural and energy-based quantities following the protocols from previous studies by us and others; see, for example, Reference [52].

**Figure 6:**
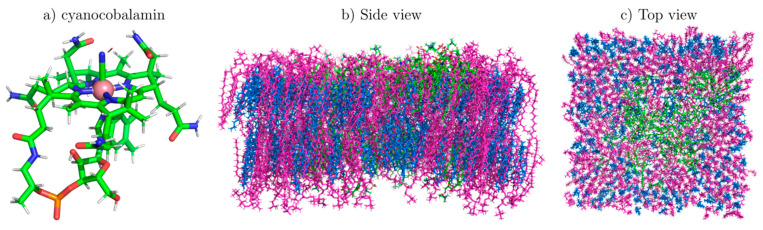
Molecular dynamic snapshots. (**a**) Cyanocobalamin and (**b**,**c**) CoPoP functionalized bilayer at the end of the 500 ns simulation (CHL1 in blue, DPPC in magenta, Co in pink and CoPoP in green). (**b**) Side view, (**c**) top view. CoPoP forms looser clusters compared to the surrounding lipids.

Figure 6 shows configurations of cyanocobalamin and the CoPoP functionalized bilayer at the end of the respective simulations. We used cyanocobalamin instead of aquocobalamin (used in the experimental studies) for the following reasons: The difficulties of parameterizing metal-containing molecules are extensively discussed by Pavlova et al. whose recently developed CHARMM36-compatible force field cyanocobalamin is used here [53]. The use of cyanocobalamin is justified since previous models show that the structures of cyanocobalamin and aquocobalamin are similar, the ligand being the principal difference [33,54]. Since we are not simulating chemical reactions but are interested in structural information, using cyanocobalamin is a good choice and avoids unnecessary and tedious force field parameterization. In addition, both the model used here [53] and the previous parameterization by Marques et al. for the Amber force field that includes cyano- and aquocobalamin (but is not compatible with CHARMM36, which is currently one of the most accurate force fields for lipids) use a consensus structure for the corrin ring [33]. Snapshots of the NTA functionalized bilayer are displayed in Figure S4 in the Supporting Information. Figure 6a shows a close-up of a cyanocobalamin molecule with its Co atom indicated in pink for clarity. Figure 6b,c contains side and top views of the DPPC-Chol-CoPoP system. Interestingly, the top view (Figure 6c) shows that the CoPoP molecules (green) self-assemble to form a cluster; initially the CoPoP molecules were distributed randomly. Although not immediately apparent in the figure, the number of cholesterols inside the CoPoP cluster is depleted as compared to areas in which no CoPoP molecules are present. This is illustrated in Figure S5 in the Supporting Information and we will return to this later when we analyze the measurements below. We note that aquocobalamin was used in the experimental studies whereas the simulations made use of cyanocobalamin as a control, see the detailed discussion in section Materials and Methods.

**Figure 7:**
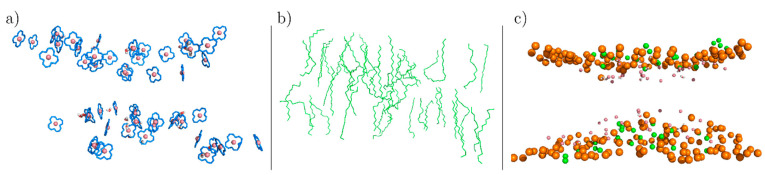
Details of the CoPoP bilayer structure. (**a**) A snapshot showing CoPoP porphyrin rings and water inclusion in the functionalized bilayer. Water molecules (in the immediate vicinity of the rings) are displayed with small red and white spheres (only a few are present), CoPoP rings in blue and the Co atoms as pink spheres. (**b**) The hydrocarbon chains of the CoPoP molecules. (**c**) The relative positions of the DPPC and CoPoP headgroups in the bilayer: Orange spheres represent the P atoms of the DPPC and green spheres the P21 atoms of the CoPoP. The smaller pink spheres represent the Co atoms. Water molecules are represented with the spheres in red and white. See Figure S2 for atom numbering.

To gain a better view of how the CoPoP molecules behave, Figure 7a shows the structures of the CoPoP rings in the bilayer and water molecules (red and white) that managed to penetrate the membrane and become proximal to the rings. Other molecules are not shown for clarity. Interactions between the Co atoms and water molecules are an important characteristic and analysis of the lifetimes of uninterrupted contacts between these water molecules and the Co atoms shows that water molecules remain inside the bilayer for extended periods of time, between 5 and 50 ps sometimes reaching up to 200 ps. Notably, these times are the contact times and the actual residence times in the CoPoP region are even longer.

The hydrocarbon chains of the CoPoP molecules are shown in Figure 7b. The figure indicates that two different populations are present: (1) well-ordered and (2) disordered tails. The presence of disorder is a key property as will be elaborated below. Figure 7c displays the relative positions of the DPPC and CoPoP headgroups. Most of the CoPoP and DPPC head groups are almost at the same height. To briefly summarize the central observations in Figure 7: (1) All the water molecules that have penetrated the membrane are either coordinated or are in the immediate vicinity of the Co atoms (Figure 7a), (2) the ring systems are clearly tilted away from the bilayer normal (Figure 7a), (3) the hydrocarbon tails of the CoPoP molecules in the central part of the cluster are disordered and appear to be in the liquid-disordered phase as compared to the gel phase of the DPPC molecules (Figure 7b), and (4) the CoPoP molecules that are at the periphery of the cluster show gel-like tails as they have not yet been fully integrated into the cluster (Figure 7b). We now analyze these properties quantitatively as discussed below.

Starting from the bilayer properties, the area per lipid and bilayer thickness are among the most common characteristics and they were calculated using the FATSLiM [55] program, which employs the Voronoi tessellation method for the area per lipid. This allows for calculation of the areas per lipid for each of the components as well as determination of local thickness. For the reference systems (non-functionalized bilayers), the component-wise average areas per lipid are 0.36 ± 0.01 nm^2^ and 0.45 ± 0.03 nm^2^ for CHL1 and DPPC, respectively. These are in good agreement with the values reported in the literature [56,57,58]. Within the functionalized bilayer, these averages are 0.38 ± 0.01 nm^2^, 0.47 ± 0.01 nm^2^, and 0.64 ± 0.02 nm^2^, for CHL1, DPPC, and CoPoP, respectively. Importantly, these results show that unlike the rest of the bilayer, the area occupied by the CoPoP cluster (see Figure 6b,c and Figure 7b) is not in the gel phase. This conclusion is clear from the area per lipid values as the values above indicate [57]. Thus, the CoPoP cluster forms a nanodomain surrounded by the binary DPPC-cholesterol matrix that remains in the gel phase. It is important to note that the CoPoP molecules were initially randomly distributed with their hydrocarbon tails ordered (they were in the gel phase); as they cluster, they undergo a transition to the liquid-disordered phase.

The presence of nanodomains provides a mechanism for water molecules to access the bilayer core: The enlarged area per lipid and the tilted orientation of the CoPoP ring system allow for a small number of water molecules to penetrate into the bilayer and to coordinate with the Co atoms as shown in Figure 7a,c. Previous studies have shown that histidine inserts spontaneously into a DPPC membrane with a free energy minimum below the lipid group [59]. It is, however, much harder for water to access the membrane interior: Sapay et al. computed the free energies of insertion of a single water molecule into various phospholipid membranes including DPPC and no minimum within the bilayer was observed [60]. Importantly, this behavior can be controlled by lipid functionalization and in our previous work we have shown that functionalizing the hydrocarbon chains with porphyrin derivatives allows for controlling the amount of water that can access the membrane interior in the membrane region that contains such modified lipids [61]. The relevance of the current results together with the previous ones as discussed above can be summarized as follows: CoPoP lipid functionalization leads to formation of nanodomains in the lipid matrix and allows for a small number of water molecules to penetrate the membrane. When histidines are present, they can, due to their favorable free energy inside the membrane, easily replace the water molecules and coordinate with the Co atoms in the CoPoP ring systems.

**Figure 8:**
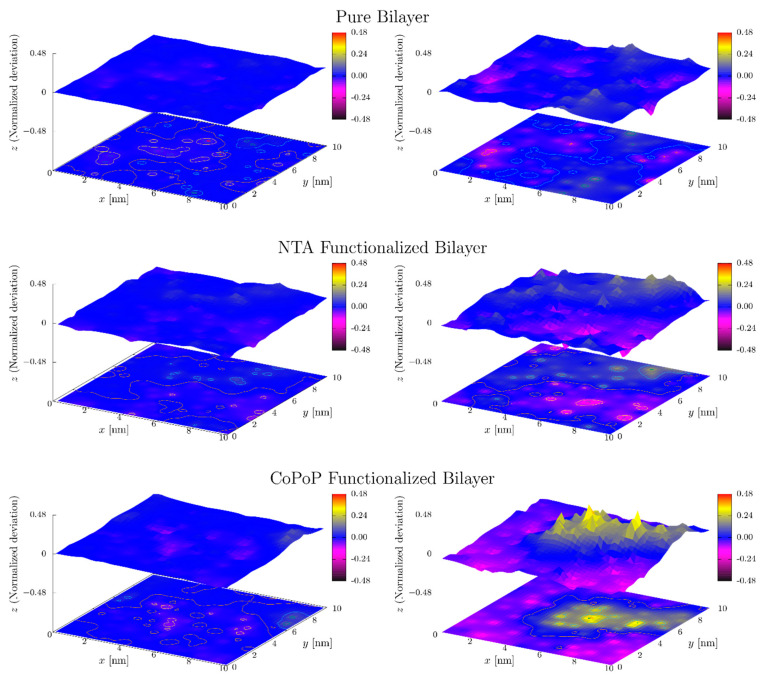
Bilayer surfaces. Fitted and smoothed surfaces of the reference system, and nitrilotriacetic acid (NTA)- and CoPoP-functionalized bilayers at the end of the respective simulations (reference dipalmitoylphosphatidylcholine (DPPC)-cholesterol system: 500 ns; NTA: 400 ns; CoPoP: 500 ns). The upper leaflets are shown in the left column and the lower ones in the right column. The z-axis of the upper figures corresponds to the normalized deviation from the mean height and the lower ones show the corresponding contours. The P atom was used as the reference for the DPPC lipids, N126 for NTA and P21 for CoPoP (see Figure S2 for atom numbering). Top: reference bilayer; middle: NTA-functionalized bilayer; bottom: CoPoP-functionalized bilayer. In the case of NTA and CoPoP, the functionalized lipids are located in the lower leaflet.

To analyze the local thickness of the bilayer, the normalized deviation from the average z-position of the headgroup was computed. The aim of this analysis is to further examine the character of the nanodomain with larger area per lipid: In the gel phase the lipid tails are extended giving rise to a thicker membrane. Figure 8 shows that the normalized deviation from the mean z-position may amount close to 50% of the height. The figure shows the existence of a thinner area that corresponds to the CoPoP cluster while the rest of the bilayer remains in the gel phase with much higher thickness. This analysis verifies the conclusion of the co-existence of the gel and liquid-disorded phases as discussed above, see also Figures S3 and S4 in the Supporting Information.

**Figure 9:**
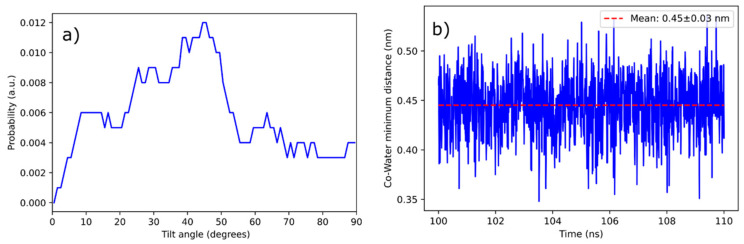
Porphyrin tilting and water access in CoPoP bilayers. (**a**) Histogram of the orientation of the porphyrin rings with respect to the bilayer normal (see Figure 7a for a snapshot). (**b**) Representative water—CoPoP minimum distance as a function of time in cyanocobalamin over 10 ns period from the middle of a trajectory.

**Figure 10:**
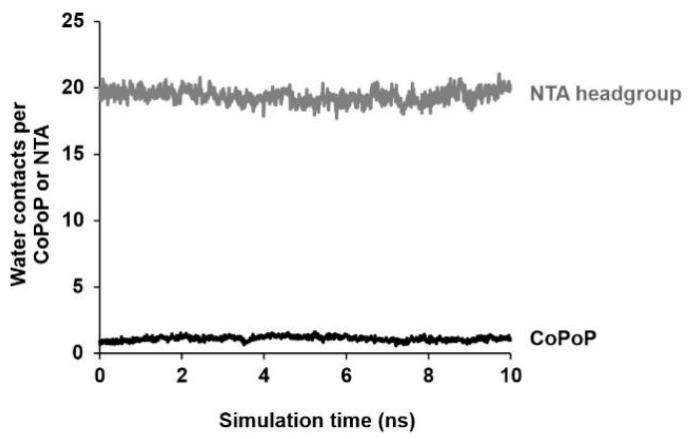
Water contact between metal centers of CoPoP or NTA within bilayers. Water contacts in the volume immediately surrounding (0.45 nm) the CoPoP cobalt or the average NTA centers. The final 10 ns of two 500 ns simulations are shown.

As discussed above, the CoPoP-rich domain has a larger area per lipid with disordered tails. The larger area per lipid allows for some water molecules to penetrate inside the bilayer. To quantify the behavior of the porphyrin rings, we analyzed their tilt angles. For the water molecules to be able to remain inside the bilayer for extended times, they have to form relatively stable contacts with the porphyrin ring systems that contain the Co atoms. The analysis shows that the porphyrin rings are tilted on average by about 45° ± 5° with respect to the bilayer normal (Figure 9a), thus allowing for the Co atoms to form strong contacts with the water molecules. As discussed above, this provides an access point for the histidine residues of the his-tagged protein to form a contact and coordinate with the cobalt; the tilted orientation of the porphyrin rings is essential for this to occur. In addition, the minimum distance between these water molecules and the Co atoms was determined to be 0.45 ± 0.03 nm (Figure 9b), and the average number of water molecules at that distance is 1.1 ± 0.2 (Figure 10) indicating significant hydration in the area where the Co atoms are located.

The experimental data in this work confirmed that Co-NTA lipids were not as effective as CoPoP with respect to serum stability. To assess the microscopic origin of this further, the data from the simulations regarding the water contact sites of the NTA headgroups, which are in the aqueous phase, were compared to the CoPoP cobalt site, which resides within the bilayer. As shown in Figure 10, the NTA headgroups had a substantially greater number of water contacts, 19.4 ± 0.5 at the distance of 0.45 nm compared to 1.1 ± 0.2 at the same distance for CoPoP. Thus, in serum conditions, it is likely that (1) water surroundsthe NTA headgroup and (2) serum proteins could compete and displace the his-tagged protein from the NTA headgroups, whereas the CoPoP does not allow sufficient access and remains serum stable.

## 4. Conclusions

Stability of antigen attachment to metallo-chelating liposome based on cobalt porphyrin phospholipid inclusion in the bilayer is superior compared to that in the non-particleized form or surface-chelated cobalt. As such, higher particle internalization by macrophages was observed with the liposome-bound Pfs25 than with the non-associated forms. The particleized cobalt tetrapyrrole also induced higher immunogenicity than the non-particleized counterpart and alum. SMFA studies demonstrated that higher levels of functional antibodies can be induced with the cobalt tetrapyrrole in the liposomal form.

We used atomistic MD simulations to establish the physical mechanism that accounts for the observed stability of the CoPoP systems. In our previous combined MD and experimental studies using porphyrin-based liposomes and membranes, we found that the rings of the porphyrin-based lipids were able to modify bilayer properties such that compactness and access of water to the membrane interior could be controlled for controlled drug release and for better MRI contrast [61,62]. The situation here is analogous in the sense that the ring-like structures of the CoPoP lipids allow for a small number of water molecules to access the membrane interior. This is different from the NTA-functionalized bilayer, which had extensive contact with water in the headgroup region. Therefore, these MD simulations imply that the limited access to water in the CoPoP bilayer enables a route for initial interaction with his-tagged proteins. This small amount of water contact also may be appropriate for shielding the cobalt-his tag from competition with serum proteins. From the physical point of view, the situation here is even more interesting as the CoPoP-functionalized membranes show the presence of nanodomains with the matrix being the gel phase and the nanodomain appears to be in the liquid-disordered phase.

## Data Availability

The data presented in this study are available on request from the corresponding author.

## Supplementary Materials

The following are available online at https://www.mdpi.com/1999-4923/13/1/98/s1, Figure S1: Gating strategy. Figure S2: Chemical structures: S3. Figure S3: Area per lipid. Figure S4: Bilayer snapshot. Figures S5: Cholesterol in bilayer.

## References

[1] Draper S.J., Angov E., Horii T., Miller L.H., Srinivasan P., Theisen M., Biswas S. (2015). Recent advances in recombinant protein-based malaria vaccines. Vaccine.

[2] Schwendener R.A. (2014). Liposomes as vaccine delivery systems: A review of the recent advances. Ther. Adv. Vaccines.

[3] Oakes R.S., Froimchuk E., Jewell C.M. (2019). Engineering Biomaterials to Direct Innate Immunity. Adv. Ther..

[4] Bookstaver M.L., Tsai S.J., Bromberg J.S., Jewell C.M. (2018). Improving Vaccine and Immunotherapy Design Using Biomaterials. Trends Immunol..

[5] Narasimhan B., Goodman J.T., Ramirez J.E.V. (2016). Rational Design of Targeted Next-Generation Carriers for Drug and Vaccine Delivery. Annu. Rev. Biomed. Eng..

[6] Wilson-Welder J.H., Torres M.P., Kipper M.J., Mallapragada S.K., Wannemuehler M.J., Narasimhan B. (2009). Vaccine adjuvants: Current challenges and future approaches. J. Pharm. Sci..

[7] Alving C.R., Rao M., Steers N.J., Matyas G.R., Mayorov A.V. (2012). Liposomes containing lipid A: An effective, safe, generic adjuvant system for synthetic vaccines. Expert Rev. Vaccines.

[8] Schmidt S.T., Foged C., Korsholm K.S., Rades T., Christensen D. (2016). Liposome-Based Adjuvants for Subunit Vaccines: Formulation Strategies for Subunit Antigens and Immunostimulators. Pharmaceutics.

[9] Nisini R., Poerio N., Mariotti S., De Santis F., Fraziano M. (2018). The Multirole of Liposomes in Therapy and Prevention of Infectious Diseases. Front. Immunol..

[10] Watson D.S., Endsley A.N., Huang L. (2012). Design considerations for liposomal vaccines: Influence of formulation parameters on antibody and cell-mediated immune responses to liposome associated antigens. Vaccine.

[11] Serre K., Machy P., Grivel J.C., Jolly G., Brun N., Barbet J., Leserman L. (1998). Efficient presentation of multivalent antigens targeted to various cell surface molecules of dendritic cells and surface Ig of antigen-specific B cells. J. Immunol..

[12] Křupka M., Mašek J., Bartheldyová E., Knötigová P.T., Plocková J., Korvasová Z., Škrabalová M., Koudelka Š., Kulich P., Zachová K. (2012). Enhancement of immune response towards non-lipidized Borrelia burgdorferi recombinant OspC antigen by binding onto the surface of metallochelating nanoliposomes with entrapped lipophilic derivatives of norAbuMDP. J. Control. Release.

[13] Shao S., Rajendiran V., Lovell J.F. (2019). Metalloporphyrin nanoparticles: Coordinating diverse theranostic functions. Co-Ord. Chem. Rev..

[14] Shao S., Geng J., Yi H.A., Gogia S., Neelamegham S., Jacobs A., Lovell J.F. (2015). Functionalization of cobalt porphyrin–phospholipid bilayers with his-tagged ligands and antigens. Nat. Chem..

[15] Bunker A., Magarkar A., Viitala T. (2016). Rational design of liposomal drug delivery systems, a review: Combined experimental and computational studies of lipid membranes, liposomes and their PEGylation. Biochim. Biophys. Acta.

[16] Huang W.-C., Deng B., Lin C., Carter K.A., Geng J., Razi A., He X., Chitgupi U., Federizon J., Sun B. (2018). A malaria vaccine adjuvant based on recombinant antigen binding to liposomes. Nat. Nanotechnol..

[17] Kumar R., Ray P.C., Datta D., Bansal G.P., Angov E., Kumar N. (2015). Nanovaccines for malaria using Plasmodium falciparum antigen Pfs25 attached gold nanoparticles. Vaccine.

[18] Kumar R., Ledet G., Graves R.A., Datta D., Robinson S., Bansal G.P., Mandal T.K., Kumar N. (2015). Potent Functional Immunogenicity of Plasmodium falciparum Transmission-Blocking Antigen (Pfs25) Delivered with Nanoemulsion and Porous Polymeric Nanoparticles. Pharm. Res..

[19] Jones R.M., Chichester J.A., Mett V., Jaje J., Tottey S., Manceva S., Casta L.J., Gibbs S.K., Musiychuk K., Shamloul M. (2013). A Plant-Produced Pfs25 VLP Malaria Vaccine Candidate Induces Persistent Transmission Blocking Antibodies against Plasmodium falciparum in Immunized Mice. PLoS ONE.

[20] Li Y., Leneghan D.B., Miura K., Nikolaeva D., Brian I.J., Dicks M.D.J., Fyfe A.J., Zakutansky S.E., De Cassan S., Long C.A. (2016). Enhancing immunogenicity and transmission-blocking activity of malaria vaccines by fusing Pfs25 to IMX313 multimerization technology. Sci. Rep..

[21] Brune K.D., Leneghan D.B., Brian I.J., Ishizuka A.S., Bachmann M.F., Draper S.J., Biswas S., Howarth M. (2016). Plug-and-Display: Decoration of Virus-Like Particles via isopeptide bonds for modular immunization. Sci. Rep..

[22] Huang W.-C., Deng B., Seffouh A., Ortega J., Long C.A., Suresh R.V., He X., Miura K., Lee S.-M., Wu Y. (2020). Antibody response of a particle-inducing, liposome vaccine adjuvant admixed with a Pfs230 fragment. NPJ Vaccines.

[23] Huang W.-C., Deng B., Mabrouk M.T., Seffouh A., Ortega J., Long C., Miura K., Wu Y., Lovell J.F. (2020). Particle-based, Pfs230 and Pfs25 immunization is effective, but not improved by duplexing at fixed total antigen dose. Malar. J..

[24] Huang W.-C., Zhou S., He X., Chiem K., Mabrouk M.T., Nissly R.H., Bird I.M., Strauss M., Sambhara S., Ortega J. (2020). SARS-CoV-2 RBD Neutralizing Antibody Induction is Enhanced by Particulate Vaccination. Adv. Mater..

[25] Mabrouk M.T., Huang W.-C., Deng B., Li-Purcell N., Seffouh A., Ortega J., Atilla-Gokcumen G.E., Long C.A., Miura K., Lovell J.F. (2020). Lyophilized, antigen-bound liposomes with reduced MPLA and enhanced thermostability. Int. J. Pharm..

[26] Federizon J., Frye A., Huang W.-C., Hart T.M., He X., Beltran C., Marcinkiewicz A.L., Mainprize I.L., Wills M.K., Lin Y.-P. (2020). Immunogenicity of the Lyme disease antigen OspA, particleized by cobalt porphyrin-phospholipid liposomes. Vaccine.

[27] Pati R., Shevtsov M., Sonawane A. (2018). Nanoparticle Vaccines against Infectious Diseases. Front. Immunol..

[28] Oyewumi M.O., Kumar A., Cui Z. (2010). Nano-microparticles as immune adjuvants: Correlating particle sizes and the resultant immune responses. Expert Rev. Vaccines.

[29] Shah R.R., O’Hagan D.T., Amiji M., Brito L.A. (2014). The impact of size on particulate vaccine adjuvants. Nanomedicine.

[30] Perrie Y., Crofts F., Devitt A., Griffiths H.R., Kastner E., Nadella V. (2016). Designing liposomal adjuvants for the next generation of vaccines. Adv. Drug Deliv. Rev..

[31] Lee S.-M., Wu C.-K., Plieskatt J., McAdams D.H., Miura K., Ockenhouse C.F., King C.R. (2016). Assessment of Pfs25 expressed from multiple soluble expression platforms for use as transmission-blocking vaccine candidates. Malar. J..

[32] Zheng S.Q., Palovcak E., Armache J.-P., Verba K.A., Cheng Y., Agard D. (2017). MotionCor2: Anisotropic correction of beam-induced motion for improved cryo-electron microscopy. Nat. Methods.

[33] Marques H.M., Ngoma B., Egan T., Brown K. (2001). Parameters for the amber force field for the molecular mechanics modeling of the cobalt corrinoids. J. Mol. Struct..

[34] Jo S., Kim T., Iyer V.G., Im W. (2008). CHARMM-GUI: A web-based graphical user interface for CHARMM. J. Comput. Chem..

[35] Lee S., Tran A., Allsopp M., Lim J.B., Hénin J., Klauda J. (2014). CHARMM36 United Atom Chain Model for Lipids and Surfactants. J. Phys. Chem. B.

[36] Jorgensen W.L., Chandrasekhar J., Madura J.D., Impey R.W., Klein M.L. (1983). Comparison of simple potential functions for simulating liquid water. J. Chem. Phys..

[37] Dykhuizen D.E., Polin D.S., Dunn J.J., Wilske B., Preac-Mursic V., Dattwyler R.J., Luft B.J. (1993). Borrelia burgdorferi is clonal: Implications for taxonomy and vaccine development. Proc. Natl. Acad. Sci. USA.

[38] Abraham M.J., Murtola T., Schulz R., Páll S., Smith J.C., Hess B., Lindahl E. (2015). GROMACS: High performance molecular simulations through multi-level parallelism from laptops to supercomputers. SoftwareX.

[39] Bussi G., Donadio D., Parrinello M. (2007). Canonical sampling through velocity rescaling. J. Chem. Phys..

[40] Parrinello M., Rahman A. (1981). Polymorphic transitions in single crystals: A new molecular dynamics method. J. Appl. Phys..

[41] Darden T.A., York D.M., Pedersen L. (1993). Particle mesh Ewald: AnN log(N) method for Ewald sums in large systems. J. Chem. Phys..

[42] Hess B. (2007). P-LINCS: A Parallel Linear Constraint Solver for Molecular Simulation. J. Chem. Theory Comput..

[43] Hess B., Bekker H., Berendsen H.J.C., Fraaije J.G.E.M. (1997). LINCS: A linear constraint solver for molecular simulations. J. Comput. Chem..

[44] Friedrich W. (2013). Vitamins.

[45] Pratt J.M. (1972). Inorganic Chemistry of Vitamin B12.

[46] Fedosov S.N., Berglund L., Fedosova N.U., Nexø E., Petersen T.E. (2002). Comparative Analysis of Cobalamin Binding Kinetics and Ligand Protection for Intrinsic Factor, Transcobalamin, and Haptocorrin. J. Biol. Chem..

[47] Butler C.C., Vidal-Alaball J., Cannings-John R., McCaddon A., Hood K., Papaioannou A., McDowell I., Goringe A. (2006). Oral vitamin B12 versus intramuscular vitamin B12 for vitamin B12 deficiency: A systematic review of randomized controlled trials. Fam. Pr..

[48] Frey P.A., Hegeman A.D. (2007). Enzymatic Reaction Mechanisms.

[49] Neale G. (1990). B12 binding proteins. Gut.

[50] Vermeulen A.N., Ponnudurai T., Beckers P.J., Verhave J.P., Smits M.A., Meuwissen J.H. (1985). Sequential expression of antigens on sexual stages of Plasmodium falciparum accessible to transmission-blocking antibodies in the mosquito. J. Exp. Med..

[51] Pradel G. (2007). Proteins of the malaria parasite sexual stages: Expression, function and potential for transmission blocking strategies. Parasitology.

[52] Javanainen M., Martinez-Seara H. (2016). Efficient preparation and analysis of membrane and membrane protein systems. Biochim. Biophys. Acta.

[53] Pavlova A., Parks J., Gumbart J.C. (2018). Development of CHARMM-Compatible Force-Field Parameters for Cobalamin and Related Cofactors from Quantum Mechanical Calculations. J. Chem. Theory Comput..

[54] Kratky C., Faerber G., Gruber K., Wilson K., Dauter Z., Nolting H.-F., Konrat R., Kraeutler B. (1995). Accurate Structural Data Demystify B12: High-Resolution Solid-State Structure of Aquocobalamin Perchlorate and Structure Analysis of the Aquocobalamin Ion in Solution. J. Am. Chem. Soc..

[55] Buchoux S. (2016). FATSLiM: A fast and robust software to analyze MD simulations of membranes. Bioinformatics.

[56] Ghysels A., Krämer A., Venable R.M., Teague W.E., Lyman E., Gawrisch K., Pastor R.W. (2019). Permeability of membranes in the liquid ordered and liquid disordered phases. Nat. Commun..

[57] Ermilova I., Lyubartsev A.P. (2019). Cholesterol in phospholipid bilayers: Positions and orientations inside membranes with different unsaturation degrees. Soft Matter.

[58] MacDermaid C.M., Kashyap H.K., DeVane R.H., Shinoda W., Klauda J.B., Klein M.L., Fiorin G. (2015). Molecular dynamics simulations of cholesterol-rich membranes using a coarse-grained force field for cyclic alkanes. J. Chem. Phys..

[59] Porasso R.D., Ale N.M., Aloia F.C., Masone D., Del Pópolo M.G., Ben Altabef A., Gomez-Zavaglia A., Diaz S.B., Vila J.A. (2015). Interaction of glycine, lysine, proline and histidine with dipalmitoylphosphatidylcholine lipid bilayers: A theoretical and experimental study. RSC Adv..

[60] Sapay N., Bennett W.F.D., Tieleman D.P. (2009). Thermodynamics of flip-flop and desorption for a systematic series of phosphatidylcholine lipids. Soft Matter.

[61] Shao S., Do T.N., Razi A., Chitgupi U., Geng J., Alsop R.J., Dzikovski B.G., Rheinstädter M.C., Ortega J., Karttunen M.M. (2017). Design of Hydrated Porphyrin-Phospholipid Bilayers with Enhanced Magnetic Resonance Contrast. Small.

[62] Carter K.A., Shao S., Hoopes M.I., Luo D., Ahsan B., Grigoryants V.M., Song W., Huang H., Zhang G.G., Pandey R.K. (2014). Porphyrin–phospholipid liposomes permeabilized by near-infrared light. Nat. Commun..

