# Supplementary material for: Experimental and Computational Observations of Immunogenic Cobalt Porphyrin Lipid Bilayers: Nanodomain-Enhanced Antigen Association
Source: Pharmaceutics. 2021 Jan 14;13(1):98. doi: 10.3390/pharmaceutics13010098 (PMC7828809; doi:10.3390/pharmaceutics13010098)
Supplement: Supplementary file 1 [file pharmaceutics-13-00098-s001.pdf]

SUPPLEMENTARY INFORMATION

Experimental and Computational Observations of Immunogenic Cobalt Porphyrin Lipid Bilayers: Nanodomain-enhanced Antigen Association

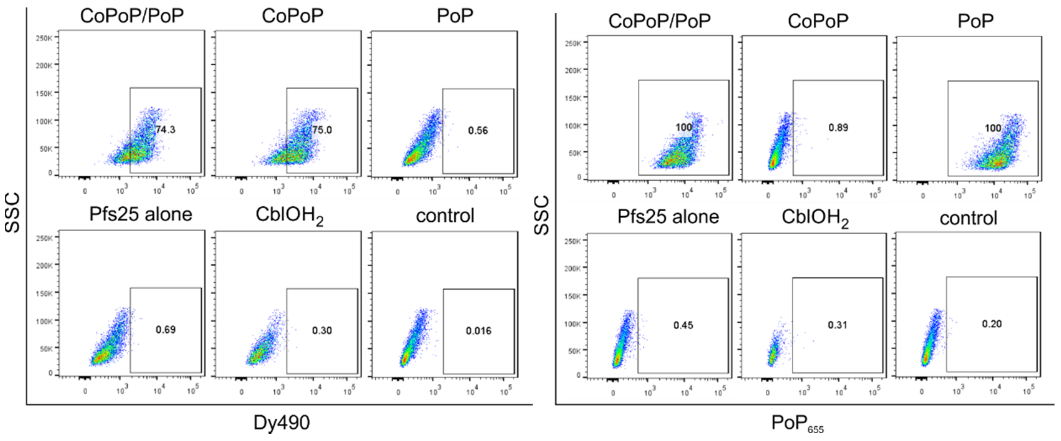

Figure S1. Gating Strategy for RAW264.1 macrophage uptake of DY-490-Pfs25 and liposomes. Cells were first gated based on the forward and side scatter. Images are representative for three different experiments.

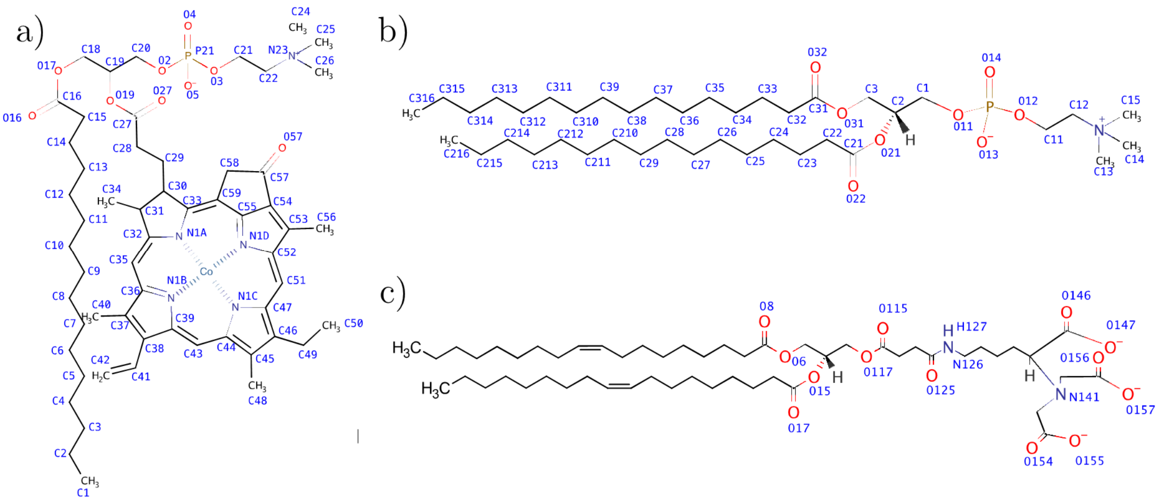

Figure S2. Chemical structures and atom numbering of the DPPC (a), CoPoP (b) and NTA (c) molecules.

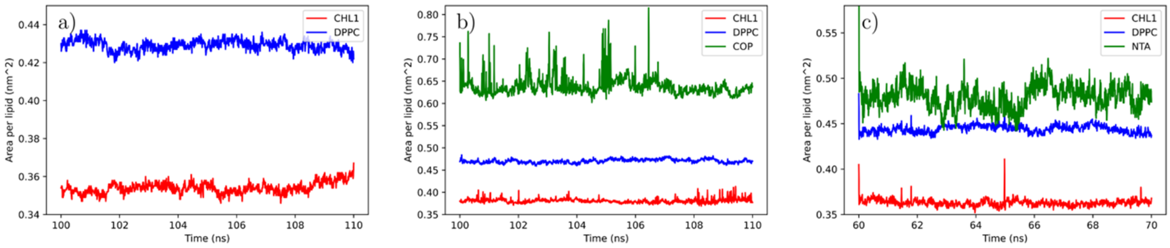

Figure S3. Area per lipid. a) Area per lipid by type for the reference bilayer. b) Area per lipid by type for the CoPoP functionalized bilayer. c) Area per lipid by type for the NTA functionalized bilayer. The spike-like fluctuations are due to the difficulty in determining area per lipid for molecules that were far away from the membrane plane (see also the Figure 8).

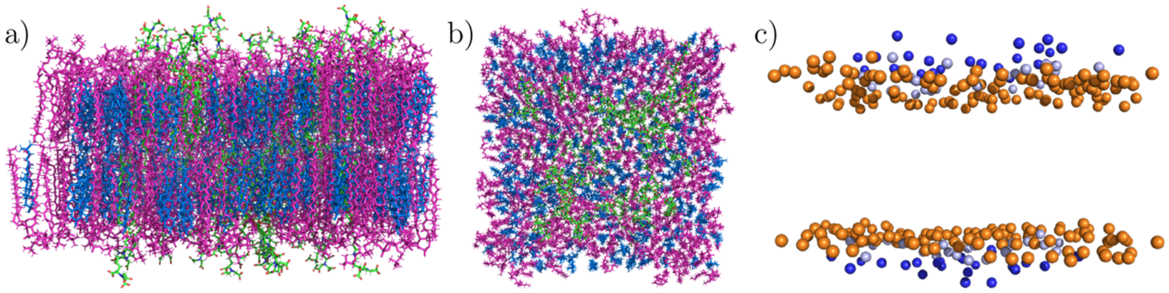

Figure S4. Snapshots of the NTA functionalized bilayer at the end of the simulation (CHL1 in blue, DPPC in magenta, and NTA in green). a) Side view, b) top view. c) The relative positions of the DPPC and NTA head groups in the bilayer: Orange spheres represent the P atoms of the DPPC. The light blue and deep blue spheres represent the N126, and N141 atoms of the NTA, respectively. The pink spheres represent the Co atoms. See Figure S3 for atom numbering.

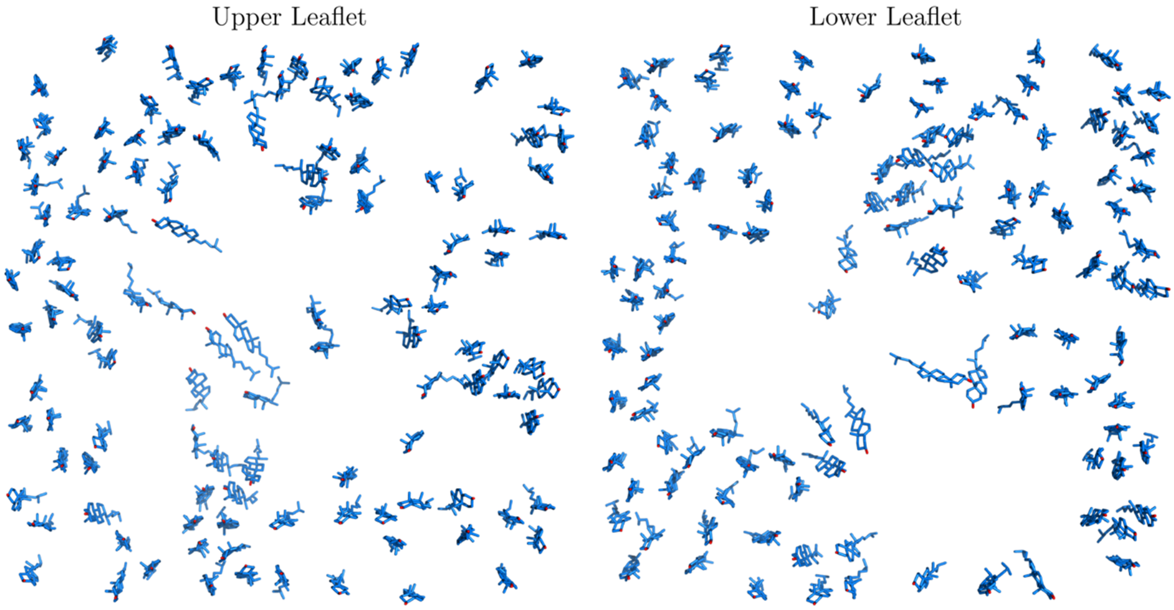

Figure S5. Top view of the cholesterol molecules within the CoPoP functionalized bilayer at the end of the simulation. Cholesterols are depleted from the areas rich with CoPoP and some of the ones located in the CoPoP rich domain have been pulled inside and their orientations become almost parallel to the membrane surface (see also Figure 6c).
